# Draft Genome Sequence of a Burkholderia cepacia Complex Strain Isolated from a Human Intra-abdominal Abscess

**DOI:** 10.1128/MRA.00091-21

**Published:** 2021-03-18

**Authors:** Yuting Zhai, Kathryn E. DeSear, Kartik Cherabuddi, J. Glenn Morris, Kwangcheol C. Jeong

**Affiliations:** a Emerging Pathogens Institute, University of Florida, Gainesville, Florida, USA; b Department of Animal Sciences, Institute of Food and Agricultural Sciences, University of Florida, Gainesville, Florida, USA; c Department of Pharmacy, University of Florida Health Shands Hospital, Gainesville, Florida, USA; d Division of Infectious Diseases and Global Medicine, College of Medicine, University of Florida, Gainesville, Florida, USA; Queens College CUNY

## Abstract

Burkholderia cepacia complex (Bcc) bacteria are opportunistic pathogens with high transmissibility and mortality. Here, we report the draft genome sequence of a Bcc strain isolated from a deep abscess culture in an immunocompetent patient with no relevant prior medical history.

## ANNOUNCEMENT

The Burkholderia cepacia complex (Bcc) is a group of opportunistic pathogens that can cause respiratory tract infections in patients with cystic fibrosis (CF) or persons who are immunocompromised (1). CF patients colonized with Bcc strains may have a life-threatening decline in lung function, leading to significantly increased mortality (2). Transmissible Bcc strains have been related to epidemic outbreaks (3). Multidrug resistance and several virulence factors are recognized as contributing to the transmissibility and persistence of B. cepacia (4, 5). In this announcement, we report the draft genome sequence of a Bcc strain, KCJ3K979, with multiple resistance genes which was isolated from a deep pancreatic abscess culture from an immunocompetent male patient in his late thirties. Bcc strains had been previously isolated over several months from various respiratory tract and blood cultures as well, with increasing resistance phenotypes. The intra-abdominal abscess ultimately resulted in the patient’s demise despite salvage treatment with meropenem-vaborbactam, piperacillin-tazobactam, and minocycline combination therapy, in addition to multiple surgical necrosectomies and washouts. Institutional review board approval was not required for this study.

KCJ3K979 was cultured overnight at 37°C in tryptic soy broth. Genomic DNA was extracted using a DNeasy blood and tissue kit (Qiagen, Valencia, CA), following the manufacturer’s instructions. Libraries were constructed with a Nextera XT sample preparation kit (Illumina, San Diego, CA), following the manufacturer’s instructions. Genome sequencing was performed using the Illumina MiSeq platform with a 2 × 250-bp, 500-cycle cartridge from MiSeq reagent kit v2 (Illumina, San Diego, CA). The total number of reads and coverage for KCJ3K979 were 669,900 and 22×, respectively. Sickle v1.33.2 (6) was used to trim the raw sequencing data, with the quality and length threshold set as 30 and 50 bp, respectively. *De novo* assembly was performed using SPAdes v3.12.0 (7), with “21,33,55,77,99,127” selected for “K-mer” and “auto” for “Coverage Cutoff.” Contigs of less than 200 bp were removed, producing a total of 526 contigs. The genome assembly quality was assessed using QUAST v5.0.2 (8). The final assembled genome size was 7,601,381 bp, with an *N*_50_ value of 29,854 bp and a GC content of 66.75%.

Multilocus sequence typing (MLST) of KCJ3K979 was performed through MLST v2.0 (9). The sequence type (ST) for KCJ3K979 was unknown, with the nearest types provided as ST830, ST832, ST466, and ST34. The sequence was annotated using the NCBI Prokaryotic Genome Annotation Pipeline (PGAP), with which a total of 7,103 coding sequences (CDS), 61 tRNAs, 4 complete rRNAs, and 4 noncoding RNAs (ncRNAs) were identified. Seven genes were predicted to encode β-lactamases, including the carbapenem-resistance gene, *bla*_OXA_. In addition, analysis of the whole-genome sequence using the Resistance Gene Identifier (RGI) v5.1.0 of the Comprehensive Antibiotic Resistance Database (CARD) v3.0.8 (10) revealed additional antibiotic resistance genes, including *adeF*, *tet*(D), and *amrA*. A total of 32 virulence factors related to antiphagocytosis, invasion, iron uptake, and signaling were recognized by PATRIC v3.6.5 as well (11).

### Data availability.

This whole-genome shotgun project has been deposited at DDBJ/ENA/GenBank under the accession number JACFYV000000000. The version described in this paper is the first version, JACFYV000000000.1. The reads are available through the NCBI Sequence Read Archive under the accession number SRR13565514.
